# Are visual impairments responsible for emotion decoding deficits in alcohol-dependence?

**DOI:** 10.3389/fnhum.2014.00128

**Published:** 2014-03-10

**Authors:** Fabien D’Hondt, Franco Lepore, Pierre Maurage

**Affiliations:** ^1^Département de Psychologie, Centre de Recherche en Neuropsychologie et Cognition, Université de MontréalMontréal, QC, Canada; ^2^Centre de Recherche CHU Sainte-JustineMontréal, QC, Canada; ^3^Laboratory for Experimental Psychopathology, Faculty of Psychology, Psychological Sciences Research Institute, Université catholique de LouvainLouvain-la-Neuve, Belgium

**Keywords:** alcohol-dependence, emotion, vision, orbitofrontal cortex, amygdala, magnocellular pathways, dorsal visual stream

## Abstract

Emotional visual perception deficits constitute a major problem in alcohol-dependence. Indeed, the ability to assess the affective content of external cues is a key adaptive function, as it allows on the one hand the processing of potentially threatening or advantageous stimuli, and on the other hand the establishment of appropriate social interactions (by enabling rapid decoding of the affective state of others from their facial expressions). While such deficits have been classically considered as reflecting a genuine emotion decoding impairment in alcohol-dependence, converging evidence suggests that underlying visual deficits might play a role in emotional alterations. This hypothesis appears to be relevant especially as data from healthy populations indicate that a coarse but fast analysis of visual inputs would allow emotional processing to arise from early stages of perception. After reviewing those findings and the associated models, the present paper underlines data showing that rapid interactions between emotion and vision could be impaired in alcohol-dependence and provides new research avenues that may ultimately offer a better understanding of the roots of emotional deficits in this pathological state.

## INTRODUCTION

Alcohol-dependence, which constitutes one of the main public health concerns worldwide (World Health Organization, 2011), is known to be associated with large-scale cerebral impairments (Bühler and Mann, 2011), notably leading to decrements in emotional abilities. Indeed, emotional deficits have been widely described (Philippot et al., 1999; Maurage et al., 2012) among alcohol-dependent individuals (ADIs) and play a crucial role in the development and maintenance of this disease (Zywiak et al., 2003; Clark et al., 2007). More specifically, these deficits have been repeatedly reported for the decoding of emotional facial expressions (EFEs; Philippot et al., 1999; Kornreich et al., 2001; Maurage et al., 2008a,b,c, 2009) and appear to be generalized to all types of emotional visual stimuli, including emotional body postures (Maurage et al., 2009). Nevertheless, while they are classically considered as indexing a genuine emotional impairment, recent results suggest that visual deficits might play a role in these emotional alterations (Maurage et al., 2007, 2008a).

The main aim of the present paper is thus to underline the importance of studying vision–emotion interactions in alcohol-dependence, as it might lead to a deep reinterpretation of earlier studies exploring emotional processing among ADI, which in turn will improve the fundamental understanding of this disease and help to develop new therapeutic approaches. To this end, we first provide a brief statement on the recent findings and models on the cerebral mechanisms sustaining emotional perception. In particular, we focus on neurophysiological data from healthy populations suggesting that mutual influences between emotion and vision start at very early stages of information processing. In relation to this, we emphasize the relevance of the “affective prediction” hypothesis (Barrett and Bar, 2009) stating that “affective responses support vision from the very moment that visual stimulation begins.” In line with this hypothesis, we suggest that, while the classical explanation of reduced visual processing of emotions in ADI focuses on emotional areas’ [amygdala, orbitofrontal cortex (OFC)] dysfunction, this impairment might also be related to alterations of early visual processing and/or connectivity between visual and emotional areas. We present a dynamic explanation according to which emotional alterations in ADI rely on a combination of these three impairments, starting with early visual deficits. Finally, we propose new research avenues to offer a better understanding of the roots of emotional deficits in alcohol-dependence.

## INTERACTIONS BETWEEN EMOTIONAL AND VISUAL PROCESSING

Cortical visual processing is classically divided into an occipito-temporal “ventral” stream mediating visual recognition and an occipito-parietal “dorsal” stream ensuring visuospatial processing, both streams having targets within the prefrontal cortex (see Kravitz et al., 2011 for a review). This segregation may originate at the retinal level: The cone photoreceptors appear to be linked to the parvocellular (PC) pathways, which slowly convey detailed (i.e., high spatial frequency, HSF) information and mainly feed into the ventral stream. Conversely, the rod photoreceptors appear related to the fast magnocellular (MC) pathways, which rapidly convey coarse (low-spatial frequency, LSF) information and mainly feed into the dorsal stream (Merigan and Maunsell, 1993; Bullier, 2001). In this framework, recent results suggest that an early affective processing of visual stimuli could rely on the extraction of coarse cues conveyed by fast MC pathways (West et al., 2010), which may mediate increased perceptual processing of emotional stimuli. For instance, studies that used event-related potentials (ERPs) revealed that negative stimuli such as unpleasant scenes (Alorda et al., 2007) or fearful faces (Pourtois et al., 2005; Vlamings et al., 2009) containing only LSF information elicit greater P1 (a positive ERP appearing around 100 ms after stimulus onset at occipito-temporal sites) amplitudes than their neutral counterparts in occipital areas, whereas no significant effects were found for equivalent stimuli containing only HSF information. This early emotional processing from LSF information may account for emotional responses occurring: (1) before the full-fledged visual processing (Vuilleumier, 2005; Rudrauf et al., 2008); (2) when the individual is engaged in a resource-consuming task (Carretié et al., 2012); and (3) when emotional stimuli are not the target of gaze (Rigoulot et al., 2011, 2012; D’Hondt et al., 2013). However, it should be noted that both the involvement of each stream and their connectivity with emotional regions might vary according to stimulus category, as shown by neuroimaging explorations and studies examining brain-damaged patients. Indeed, differential occipito-temporal regions are activated during the processing of specific emotional stimuli [faces (Barton, 2003; Pitcher et al., 2008), body postures (Moro et al., 2008; Candidi et al., 2011)], and fronto-parietal networks are also involved in the simulation of observed facial and body movements (Urgesi et al., 2007; Avenanti et al., 2013). The visuo–emotional interactions described below might thus be influenced by variations in the type and movement of the stimuli.

One proposal that has predominated for a decade suggests that the amygdala, a key region for emotional visual perception (Sergerie et al., 2008; Sabatinelli et al., 2011), ensures this early emotional processing from coarse visual cues and then sends feedback signals to the ventral visual stream (Vuilleumier, 2005). The amygdala would be responsible for enhancing the perceptual processing of emotional stimuli in the visual cortex, via bilateral connections with occipito-temporal areas (Amaral et al., 2003). Two main models explain how crude visual information reaches the amygdala (Vuilleumier, 2005): The first suggests a rapid sequential processing along the ventral visual stream that affords rapid amygdala activation and subsequent feedback signals before complete processing of the stimulus; The second implies an additional subcortical pathway to the amygdala, involving the superior colliculus and pulvinar, that would account for the very fast (Luo et al., 2007, 2010), coarse (Vuilleumier et al., 2003; Maratos et al., 2009) and non-conscious (Whalen et al., 1998; Garrido et al., 2012) visual processing of emotional stimuli. In particular, an fMRI study by Whalen et al. (2004) suggests that the activity within the ventral amygdala [i.e., the locus of convergence for most (sub)cortical inputs in the amygdaloid system] is enhanced in response to LSF information (fearful versus happy eyes). Interestingly, Rudrauf et al. (2008) compared several sequential and parallel models to determine which one would best predict magnetoencephalographic responses in participants presented with arousing visual stimuli. Their results supported the models including parallel short-cut pathways by which visual information directly reaches the “anterior affective system,” i.e., the temporal pole, amygdala, and OFC. More precisely, these models were the only ones able to account for early responses in the anterior affective system and early modulation of ventral visual processing by emotional stimuli. However, the results were comparable between models that included a direct subcortical pathway to the amygdala and others relying on cortico-cortical long-range fasciculi, i.e., (1) the inferior longitudinal fasciculus, connecting early visual cortices with the amygdala and temporal pole; (2) the inferior frontal–occipital fasciculus connecting early visual cortices with OFC (Catani et al., 2002, 2003). This latter result helps to overcome the debate whether a subcortical pathway reaching directly the amygdala is functional or not in primates (Pessoa and Adolphs, 2010; Tamietto and de Gelder, 2010). In fact, prefrontal regions, including OFC, also rapidly respond to coarse features (Bullier, 2001), which could allow a top-down facilitation of visual recognition (Bar, 2003). Future studies should thus investigate whether some long-range fasciculi directly connecting early visual regions with the OFC contain a substantial proportion of MC fibers (Rudrauf et al., 2008).

**FIGURE 1:**
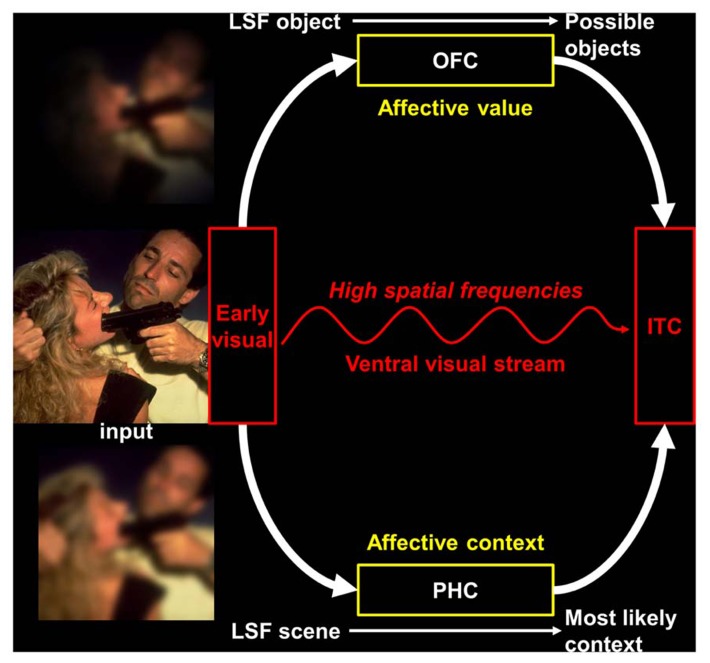
**An illustration of the affective prediction model extended to the visual scene recognition.** The image shown in this figure has been selected in the International Affective Picture System (IAPS; Lang et al., 2008). LSF, low spatial frequencies; OFC, orbitofrontal cortex; PHC, parahippocampal cortex; IT, inferior temporal cortex. Figure adapted from Bar et al. (2006), Bar (2009).

An alternative proposal was formulated by Barrett and Bar (2009), namely the affective prediction hypothesis (see **Figure 1**). This hypothesis relies on previous works (Bar, 2003; Bar et al., 2006; Kveraga et al., 2007; Barrett and Bar, 2009) showing that the OFC receives coarse visual cues before an object is recognized, and uses it to generate a prediction about object identity, which is then back-projected to occipito-temporal visual regions and promotes recognition. This facilitation of visual recognition would rely on fast MC projections connecting early visual and infero-temporal cortices with the OFC (Kveraga et al., 2007). As neuroanatomical connections from visual areas, other affective regions (amygdala, insula) and autonomic centers converge into the OFC, the latter would constitute the crucial area for vision–emotion interactions (Barrett and Bar, 2009). More precisely, the affective prediction hypothesis postulates that the different parts of the OFC would have distinct roles during visual processing. First, the medial parts would apprehend the affective significance before the stimulus is consciously perceived and prepare the perceiver to act by: (1) providing a basic-level affective prediction, on the basis of LSF information received via fast dorsal MC pathways, to modify the perceiver’s bodily state and re-create the affective context in which the stimulus was experienced in the past; (2) relaying this initial affective estimate to the lateral parietal areas of the dorsal visual stream, which is involved in spatial localization and visually guided action (Goodale and Milner, 1992). Second, the lateral parts of the OFC, which have robust reciprocal connections with inferior temporal areas of the ventral visual stream, would integrate the information about affective-based bodily changes received from the insula (Craig, 2009) with a more detailed representation of the visual input provided by HSF information that is conveyed by PC pathways (Merigan and Maunsell, 1993). The resulting multimodal representation (including information from the five senses) then influences the processing in the ventral visual stream in such a way that the conscious percept includes the affective value of the stimulus. Thus, the affective prediction model accounts for the data suggesting that a coarse but fast analysis of visual information would trigger rapid emotional processing and top-down facilitation of visual object recognition. In addition, a fast LSF image of a visual scene would also trigger a contextual facilitation for the recognition of the objects contained in this scene (Bar, 2004, 2009). Context-based predictions, notably involving the parahippocampal cortex (PHC; Bar and Aminoff, 2003; Bar, 2004), would be projected to the infero-temporal cortex. Since the activity of the PHC in response to visual scenes is known to be modulated by their affective content (Lane et al., 1997; Aldhafeeri et al., 2012), the affective prediction model should also include context-based affective predictions (see **Figure 1**). As a whole, this would provide a heuristic theoretical framework to understand how emotional processing could be intrinsically linked to visual processing.

## EMOTIONAL AND VISUAL DEFICITS IN ALCOHOL-DEPENDENCE

Up to now, the neurophysiological substrates of emotional visual perception deficits among ADI remain largely unexplored. The few studies conducted have shown that alcohol-dependence is associated with a volume loss of amygdala (Durazzo et al., 2011), which presents reduced reactivity to threat signals (Gilman and Hommer, 2008) and responds indifferently to all EFE (Marinkovic et al., 2009). In addition, a reduced insular activation has been observed during EFE decoding (O’Daly et al., 2012). In addition, there is evidence of reduced cerebral blood flow (Catafau et al., 1999) and volume loss of the OFC (Durazzo et al., 2011), which is proportional to alcohol-dependence duration (Miguel-Hidalgo et al., 2006) and predicts future relapses (Cardenas et al., 2011). A reduced activation of the OFC during EFE decoding has also been shown in ADI (O’Daly et al., 2012). Another study showed that, unlike healthy controls, ADI did not exhibit increased activation in the inferior frontal gyrus in response to EFE and also presented a blunted rostral anterior cingulate cortex response during negative EFE decoding (Salloum et al., 2007).

Several ERP studies have also been carried out to determine the stage at which observed deficits in cerebral processing among ADI originates. These studies have shown impairments (in amplitude and latency; Porjesz and Begleiter, 2003; Hansenne, 2006) of the P300, a long-lasting positive deflection appearing around 300 ms after stimulus onset and related to decisional processes (Polich, 2004). Also, during a task requiring EFE detection among a succession of neutral faces, ADI showed alterations for early visual (P1) and visual expertise (N170) stages (Maurage et al., 2007). Moreover, other studies have found that alcohol-dependence leads to delayed latency (Cadaveira et al., 1991; Nazliel et al., 2007), reduced amplitude (Chan et al., 1986; Ogura and Miyazato, 1991) and abnormal topography (Miyazato and Ogura, 1993) of the P1. The reduced abilities of ADI in emotional processing of visual stimuli are thus likely to originate at early perceptual steps.

In agreement with this, deficits in visuospatial processing (Beatty et al., 1996; Sullivan et al., 2002; Fama2004 et al., 2004) count among the most severe dysfunctions observed in recently detoxified ADI and persist for years after detoxification in some patients (Fein1990 et al., 1990). In some cases, it has been proposed that ADI use a different strategy than healthy controls when performing a specific task. For instance, it appears than ADI use higher-order cognitive processes to perform a perceptual learning task at normal levels whereas healthy controls use basic visuospatial processes (Fama et al., 2004). Furthermore, while healthy individuals use the dorsal stream for visuospatial working memory processing, ADI recruit the ventral visual stream and declarative systems to perform the same task with equivalent behavioral performance (Pfefferbaum et al., 2001; Tapert et al., 2001). Since the dorsal stream is mainly fed by LSF information conveyed by MC pathways, this could reveal a strategy change to compensate for MC deficits.

While this proposal has not been directly tested in ADI, several data suggest that alcohol consumption impairs MC pathways (Zhuang et al., 2012; Weber et al., 2013). For instance, moderate acute alcohol intake reduces temporal contrast sensitivity at high temporal frequency (Pearson and Timney, 1998) and slows down neural processing and transmission speed (Khan and Timney, 2007). Moreover, alcohol affects visual sensorimotor functions and visual fields but not other visual functions (Hill and Toffolon, 1990). To explain these results, the authors postulated higher alcohol sensitivity of (1) the rod photoreceptors in peripheral retina than the cone photoreceptors in central retina and/or (2) the dorsal visual stream than the ventral one. Otherwise this could also reveal higher alcohol sensitivity of the MC pathways than PC pathways, as the former may be mainly related to the peripheral retina and rapidly conveys LSF information to the dorsal stream (Livingstone and Hubel, 1987, 1988; Merigan and Maunsell, 1993; Lee et al., 1997; Bullier, 2001; Stephen et al., 2002).

Finally, data showing white matter abnormalities in ADI (Chanraud et al., 2009; Yeh et al., 2009; Schulte et al., 2010), which are correlated with higher relapse rates (Sorg et al., 2012), suggest that alcohol-dependence may be associated with disrupted connectivity between visual and affective brain regions. For instance, ADI show microstructural alterations of the cingulate bundle of the limbic system, suggesting possible connectivity impairments between OFC and parietal areas of the dorsal stream as well as amygdala. Moreover, a recent study revealed reduced functional connectivity between areas of the ventral visual stream and frontal regions in ADI during EFE categorization (Maurage et al., 2013). These data suggest that the classical explanation in terms of impaired emotional regions cannot fully account for the emotional deficits in ADI. We rather propose a dynamic explanation, starting at early visual processing stages and then extending during following steps, due to reduced visual-emotional connectivity and impaired emotional regions. The affective prediction model could thus account for emotional deficits in ADI.

## PERSPECTIVES AND CONCLUSION

On the basis of data reviewed above and questions raised, the main research avenues that should be addressed in the following years will now be described, by showing how alcohol-dependence may constitute a relevant psychopathological model to test the affective prediction model, and conversely how this model could help to understand the roots of emotion decoding deficits among ADI.

### MC VERSUS PC PATHWAYS

The relative magnitude of emotional processing deficits as a function of PC versus MC pathways should be investigated among ADI (notably by using ERPs). This could be done by comparing emotional processing of LSF- versus HSF-biased, and/or peripherally- versus centrally presented (as foveal and peripheral vision may be mainly related to PC and MC pathways respectively; Livingstone and Hubel, 1987, 1988; Stephen et al., 2002) stimuli. Particularly, we hypothesize that ERPs will be impaired in ADI for LSF stimuli, as indexed by the reduction or absence of two central effects observed among healthy controls, i.e., (a) shorter latency of the visual expertise component (N170) for LSF- compared to HSF-biased faces; (b) increased amplitude of the early visual components (P100 and N170) for emotional versus neutral stimuli specifically for LSF-biased stimuli. Importantly, these stimuli should be matched for luminance and contrast (Vlamings et al., 2009) to avoid any influence of low-level visual factors on the results.

### GENERALIZED VISUAL DEFICIT

Further studies in ADI should investigate the spatiotemporal stages of cerebral activity associated with the processing of various types of emotionally laden stimuli. If visual impairments are responsible for emotion decoding deficits among ADI, the latter should not be limited to specific visual stimuli such as EFE and body postures and could also be observed for other stimuli such as objects. As underlined above, visual–emotional interactions impairments might also be affected by variations in stimulus category (relying on different occipito-temporal regions) and movement (involving fronto-parietal networks).

### AFFECTIVE PREDICTION HYPOTHESIS

There is a lack of direct experimental evidence to confirm this proposal (Barrett and Bliss-Moreau, 2009). Since alcohol-dependence may be associated with alterations of dorsal MC pathways and OFC and putatively altered connectivity between OFC and visual regions, emotion decoding deficits among ADI could be due to impaired affective prediction processing. Moreover, as suggested above, the question whether context-based predictions are emotionally laden needs to be addressed. Experimental evidence could be obtained by showing that the atrophy of the PHC (Suzuki et al., 2010) and associated white matter areas (Jang et al., 2007) in alcohol-dependence induce defects in context-based affective predictions. Neuroimaging studies investigating these hypotheses as well as structural and functional connectivity between affective and visual regions in ADI are needed.

To conclude, such explorations might have crucial fundamental implications in: (1) cognitive psychology and neurosciences, by exploring the interactions between visual and emotional processes and by testing for the first time the affective prediction model in a clinical population; (2) clinical psychology and psychiatry, by offering a better understanding of the roots of emotional deficits among ADI and by paving the way for further therapeutic prospects.

## Conflict of Interest Statement

The authors declare that the research was conducted in the absence of any commercial or financial relationships that could be construed as a potential conflict of interest.
